# P-414. Needs-Based Assessment of Antimicrobial Stewardship Curriculums by Pediatric Infectious Diseases Fellowship Directors

**DOI:** 10.1093/ofid/ofaf695.631

**Published:** 2026-01-11

**Authors:** Kristin Patrick, David Haslam, Mark Murphy, Justin Markham

**Affiliations:** Johns Hopkins Hospital, Towson, MD; Cincinnati Children's Hospital Medical Center, Cincinnati, OH; Cincinnati Children's Hospital Medical Center, Cincinnati, OH; Cincinnati Children's Hospital Medical Center, Cincinnati, OH

## Abstract

**Background:**

Antimicrobial Stewardship (AS) is a crucial aspect of a pediatric ID fellow’s training to address the public health crisis of antimicrobial resistance. While adult ID programs have noted an educational gap, there is minimal information known about the status of AS fellowship education within pediatric ID training.

**Methods:**

A cross-sectional survey was created to address the current state of training and identify the current strengths and weaknesses in pediatric AS education. The survey assesses the presence of a curriculum, satisfaction with current training, current learning objectives, and barriers.

**Results:**

27 of 65 programs responded to the survey, with 63% of those that responded having a formal AS curriculum and all programs stating that a standardized curriculum would be useful. There was a divergence between satisfaction in fellows’ training and perceived fellows’ ability to assume a leadership role in antimicrobial stewardship post training. Some notable gaps in education included psychosocial factors, data analytics, antibiotic timeouts, and antibiotic allergy assessments. The most common barrier identified was insufficient time from an educator standpoint.

**Conclusion:**

To address the educational gap, aspiring fellows in AS should allocate dedicated time for both practical experience and scholarly pursuits. Our results identified areas to improve, which will aid in our goal to create a curriculum that builds a foundation throughout fellowship and establishes the necessary career skills to become a successful antimicrobial steward. Future steps could prioritize collaboration between AS programs to aid in the creation of a standardized antimicrobial stewardship curriculum.

**Disclosures:**

All Authors: No reported disclosures

